# Resistance training regulates gene expression of molecules associated with intramyocellular lipids, glucose signaling and fiber size in old rats

**DOI:** 10.1038/s41598-017-09343-6

**Published:** 2017-08-17

**Authors:** Manoel Benício Teixeira Ribeiro, Vinicius Guzzoni, Jeffrey M. Hord, Giselle Nunes Lopes, Rita de Cássia Marqueti, Rosângela Vieira de Andrade, Heloisa Sobreiro Selistre-de-Araujo, João Luiz Q. Durigan

**Affiliations:** 1 College of Physical Education, University of Brasília, Distrito Federal, Brazil; 2 Postdoctoral Fellowship, University of Brasília, Distrito Federal, Brazil; 3 Department of Molecular Physiology and Biophysics, Carver College of Medicine, University of Iowa, Iowa City, United States; 4 Department of Physiological Sciences, Center of Biological and Health Science, Federal University of São Carlos, São Carlos, Sao Paulo, Brazil; 5 Graduate program of Rehabilitation Sciences, University of Brasilia, Distrito Federal, Brazil; 6 Graduate program of Genomics and Proteomics, Catholic University of Brasilia, Distrito Federal, Brazil

**Keywords:** Gene expression, Ageing

## Abstract

Sarcopenia is a complex multifactorial process, some of which involves fat infiltration. Intramyocellular lipid (IMCL) accumulation is postulated to play a role on sarcopenia during aging, which is believed to be due alterations in glucose homeostasis in the skeletal muscle. Sarcopenia, along with intramuscular lipids, is associated with physical inactivity. Resistance training (RT) has been indicated to minimize the age-induced muscle skeletal adaptations. Thus, we aimed to investigate the effects of RT on mRNA levels of regulatory components related to intramyocellular lipid, glucose metabolism and fiber size in soleus and gastrocnemius muscles of aged rats. Old male rats were submitted to RT (ladder climbing, progressive load, 3 times a week for 12 weeks). Age-induced accumulation of IMCL was attenuated by RT, which was linked to a PPARy-mediated mechanism, concomitant to enhanced regulatory components of glucose homeostasis (GLUT-4, G6PDH, Hk-2 and Gly-Syn-1). These responses were also linked to decreased catabolic (TNF-α, TWEAK/Fn14 axis; FOXO-1, Atrogin-1 and MuRF1; Myostatin) and increased anabolic intracellular pathways (IGF-1-mTOR-p70S6sk-1 axis; MyoD) in muscles of trained aged rats. Our results point out the importance of RT on modulation of gene expression of intracellular regulators related to age-induced morphological and metabolic adaptations in skeletal muscle.

## Introduction

Sarcopenia is a complex multifactorial process, involving fat infiltration^1^ and a reduction in skeletal muscle cross sectional area (CSA)^2^. Intramyocellular lipid (IMCL) accumulation is postulated to play a role in the progression of sarcopenia with aging^3^. Evidence indicates that IMCL accumulation blunts muscle glucose transport activity and glycogen synthesis^4^. Accordingly, age-induced changes in mitochondrial biogenesis may affect fatty acid oxidation and result in accumulation of lipids in skeletal muscle cells leading to an alteration in glucose uptake and glycogen synthesis^5^. However, the mechanisms those mediate IMCL and glucose homeostasis during age-related muscle loss have yet to be elucidated.

Various transcription factors and intracellular pathways have been implicated in the regulation of fat and glucose metabolism. For example, peroxisome proliferator-activated receptor γ (PPARγ) and CCAAT/enhancer binding proteins (C/EBPs), such as C/EBPα, are key early regulators of adipogenesis^6^. PPAR-γ also regulates lipogenesis in skeletal muscle^7^. Additionally, PGC-1α, a PPARγ binding protein plays a role in the transcriptional control of oxidative metabolism^8^ and fiber type switching^9^. PGC-1α also increases lipogenesis and lipid catabolism in skeletal muscle^10^. Another factor is known as lipoprotein lipase (LPL), which is a key enzyme responsible for fatty acid and lipoprotein metabolism in muscle^11^. The regulation of age-related alterations in glucose and fat metabolism have been documented in mice^12^. However, the role of key regulatory components of glucose homeostasis, such as glycogen synthase type 1 (Gly-Syn-1), glucose-6-phosphate dehydrogenase (G6PDH), hexokinase type 2 (Hk-2) and glucose transporter 4 (GLUT-4) with aging is not fully understood. Considering the complexity of the crosstalk between adipogenic transcriptional factors, glycogen metabolism and atrophy/hypertrophy signaling pathways, a better understanding of molecular pathways that regulate the aging-induced phenotypes is needed.

Skeletal muscle atrophy is complex process that is due in part to proinflammatory cytokines, which includes TNF-α (tumor necrosis factor-α), TWEAK (tumor necrosis factor apoptosis inducing) and its receptor, Fn14 (growth factor-inducible 14 receiver fibroblasts)^13^. Further downstream, activation of forkhead box protein O1 (FOXO-1) promotes the expression of E3 ubiquitin ligases, Atrogin-1 and muscle ring finger protein-1 (MURF-1), which are key players in the ubiquitin-proteasome system^14^. The loss of muscle mass during aging is also under influence of various growth factors, such as those in the TGF-β (transforming growth factor-β) family, mostly notably, myostatin^15^. Along with elevated rates of protein degradation, sarcopenia has also been associated with a reduction in muscle protein synthesis^16^. Anabolic signaling is primarily attributed to the activation of anabolic signaling axis which includes IGF-1 (Insulin-like growth factor 1), mTOR (mammalian target of rapamycin) and activation of p70S6K-1 (p70S6 kinase 1)^17^. Furthermore, altered sensitivity of satellite cells is implicated in sarcopenia. Satellite cells are regulated by myogenic regulatory factors, such as MyoD that promote differentiation of the muscle-specific stem cells^18^.

Resistance training (RT) has been consistently recommended to minimize the age-related muscle adaptations^19^. In this regard, sarcopenia, along with fatty acid infiltration, is associated with physical inactivity^20^. Moreover, it has emerged that exercise training confers beneficial effects on glucose homeostasis^21^ and modulates IMCL^22^. However, the effects of RT on molecules related to glucose homeostasis and lipogenesis in skeletal muscle with advancing age are unknown. Furthermore, how RT affects intracellular signaling and transcription factors that control glucose homeostasis, lipogenesis and morphology of skeletal muscle in the aging is not understood.

Based on our assumptions, we hypothesized that RT could protect against the age-induced IMCL accumulation, glucose homeostasis and muscle atrophy via downregulation of adipogenic factors (CEPB-α, LPL, PPARγ and PGC-1α,) and atrophy-associated molecules (TNF-α, TWEAK/Fn14 axis; FOXO-1, Atrogin1, MURF-1 and myostatin) concomitant to increases in hypertrophy-associated factors (IGF-1/mTOR/p70S6k-1/MyoD) and glucose homeostasis (Gly-Syn-1, G6PDH, GLUT-4, Hk-2). Thus, we sought to investigate the effects of RT on mRNA levels of atrophy/hypertrophy signaling, intracellular fatty infiltration and glucose metabolism in the soleus and gastrocnemius muscles of aged rats.

## Results

### Body weight in old and trained rats

The iBW and fBW are shown in Table 1. The iBW of old rats was higher than young groups at the beginning of experiment - first day of RT session. After 12 weeks of RT, fBW of YS and YT rats were 57.8% and 44.4% higher in comparison to their matched iBW. On the other hand, fBW of OS and OT groups decreased (6.8% and 15%, respectively) when compared to their matched iBW.

**Table 1:** Body weight (BW), percentage of gain of BW, gastrocnemius (GAS) and soleus (SOL) weights, glycogen content of GAS and SOL muscles from experimental groups.

	YS	YT	OS	OT
iBW (g)	295.6 ± 34.6	301.8 ± 30	508.3 ± 76.6^**a,b**^	527.3 ± 75.9^**a,b**^
fBW (g)	508.3 ± 85.6	434 ± 52.7^**a**^	452 ± 86.4^**a**^	480.4 ± 66.5^**a,b,c**^
Gain (%)	57.8	44.4	−6.8	−15
GAS weight (mg)	1.190 ± 0.1	1.121 ± 0.1	1.072 ± 0.1	0.976 ± 0.1
SOL weight (mg)	0.279 ± 0.1	0.248 ± 0.02	0.237 ± 0.04	0.260 ± 0.02
GAS glycogen (mg/100 mg)	15.8 ± 0.4	23.2 ± 0.6^**a**^	15.1 ± 0.3	22.5 ± 0.5^**c**^
SOL glycogen (mg/100 mg)	11.3 ± 0.3	23.8 ± 0.3^**a**^	8.8 ± 0.4	23.1 ± 0.4^**c**^
Liver glycogen (mg/100 mg)	29 ± 2.0	58.4 ± 1.8^**a**^	33.6 ± 1.9	60 ± 1.9^**c**^

iBW = initial body weight; fBW: final body weight; Gain = percentage of increasing or decreasing of BW after 12 weeks; GAS weight = Glycogen content of gastrocnemius; SOL weight = Glycogen content of soleus. Groups: Young Sedentary (YS), Young Trained (YT), Old Sedentary (OS) and Old Trained (OT). Values are expressed as mean ± SEM. Two-way ANOVA, p < 0.05: ^a^vs. YS; ^b^vs. YT; ^c^OS. n = 6/group.

### CSA and IMCL and glycogen content of gastrocnemius (GAS) and soleus (SOL) muscles in old and trained rats

**Figure 1:**
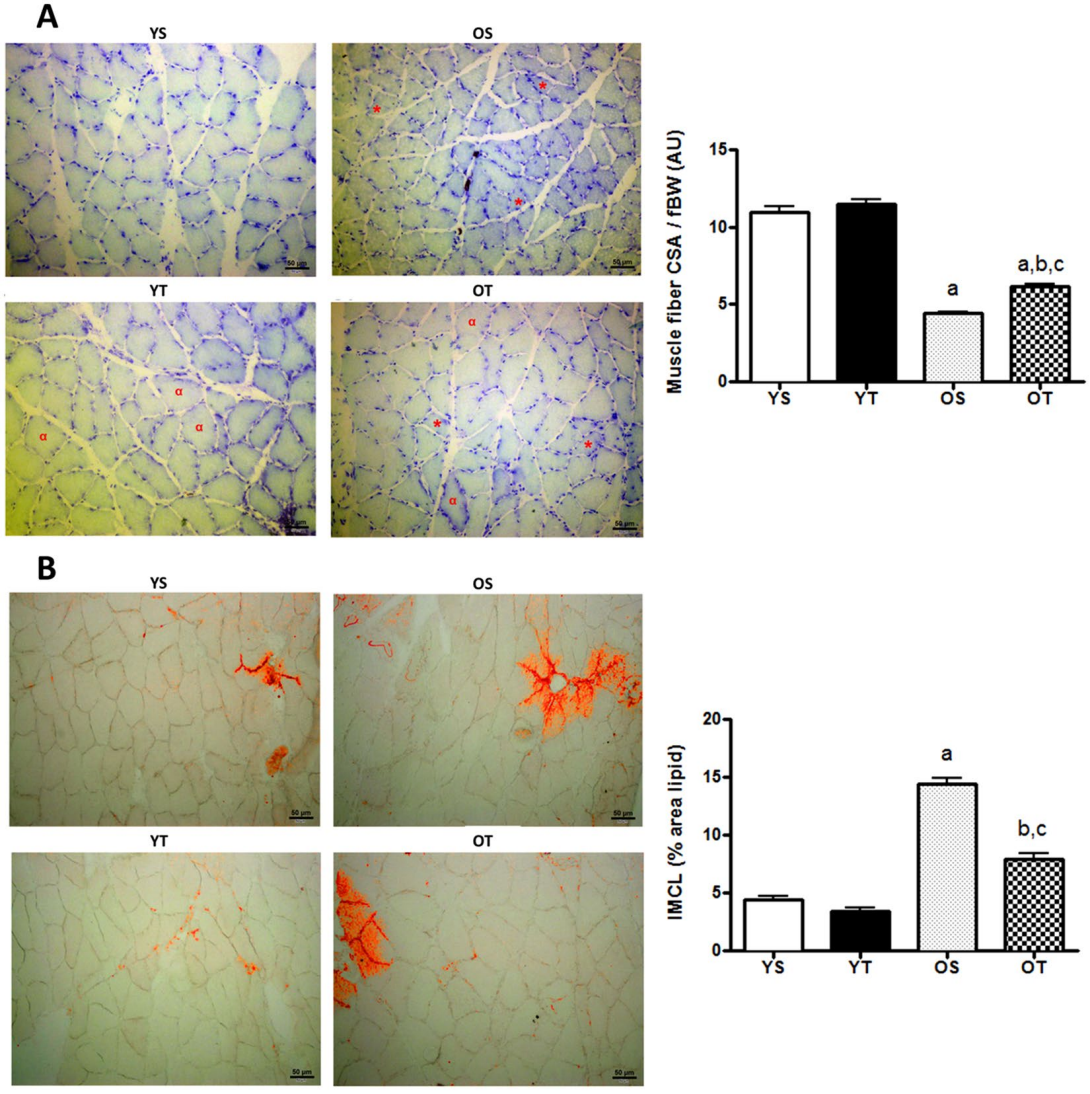
Light microscope images of hematoxilin-eosin stained sections of SOL muscle at 10X magnification. * and ^α^ means atrophied and hypertrophied fibers respectively (**A**). Quantification of cross sectional area (CSA) normalized by fBW (**B**). Light microscope images of intramyocellular lipids (IMCL) of SOL muscle at 20X magnification (**C**). Quantification of IMCL content (**D**). Groups: young sedentary (YS), young trained (YT), old sedentary (OS) and old trained (OT) rats. Values are expressed as means ± SEM. Two-way ANOVA, p < 0.05: ^a^vs. YS; ^b^vs. YT; ^c^vs. OS. n = 6/group.

**Figure 2:**
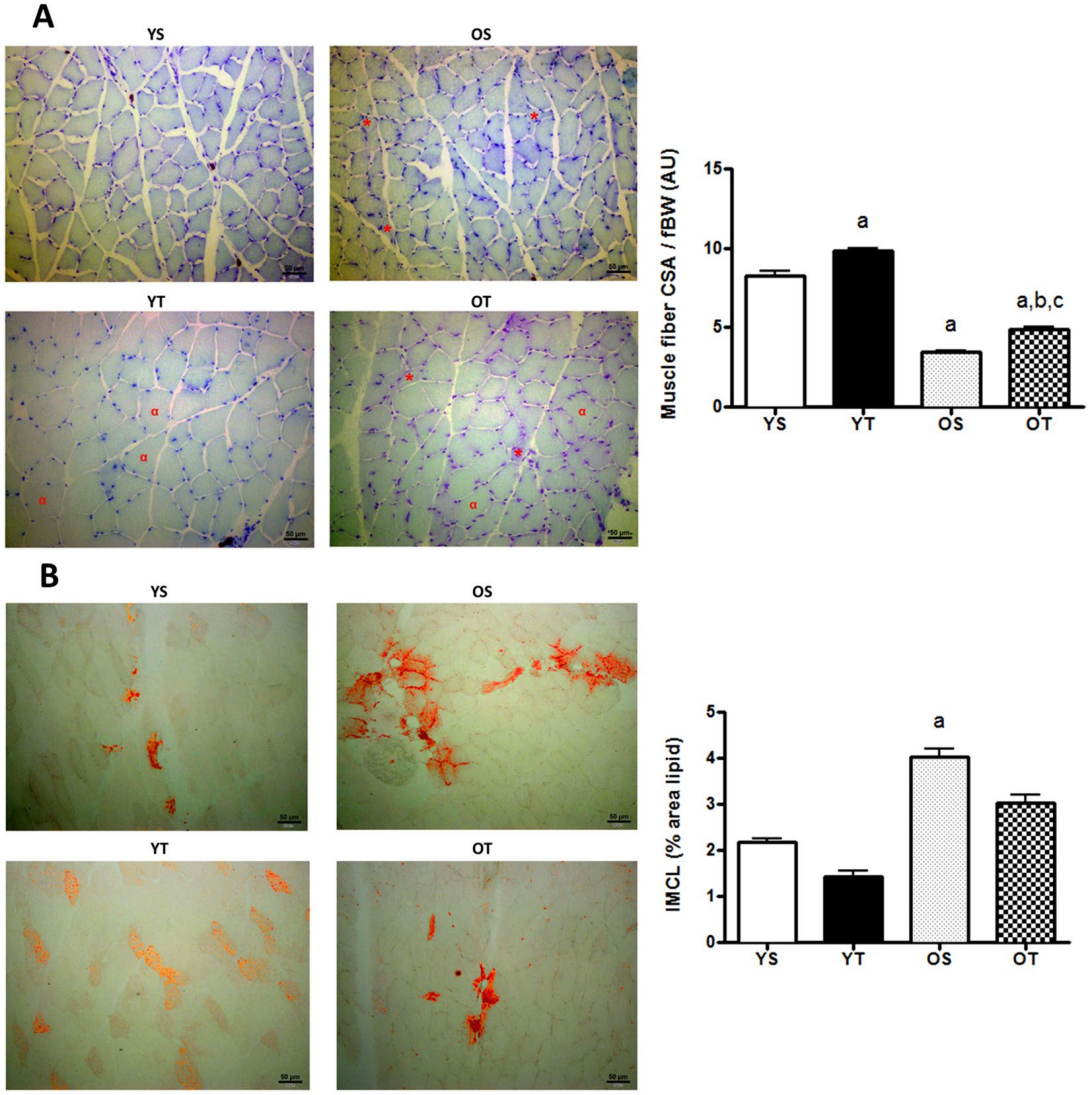
Light microscope images of hematoxilin-eosin stained sections of GAS muscle at 10X magnification. ^*^ and ^**α**^means atrophied and hypertrophied fibers respectively (**A**). Quantification of cross sectional area (CSA) normalized by fBW (**B**). Light microscope images of intramyocellular lipids (IMCL) of GAS muscle at 20X magnification (**C**). Quantification of IMCL content (**D**). Groups: young sedentary (YS), young trained (YT), old sedentary (OS) and old trained (OT) rats. Values are expressed as means ± SEM. Two-way ANOVA, p < 0.05: ^a^vs. YS; ^b^vs. YT; ^c^vs. OS. n = 6/group.

RT alleviated the age-induced decrease of CSA in SOL and GAS muscles (OT *vs*. OS) (Figs 1A and 2A), as well as the age-related increase of IMCL content in SOL muscle (Fig. 1B). Unlike SOL, CSA of GAS was larger in YT than in YS rats (Fig. 2A) and IMCL content was greater in OS than in YS group (OS *vs*. YS), with no effect of RT (Fig. 2B). On the other hand, RT enhanced tissue glycogen content in SOL and GAS, in both young and old groups (OT *vs*. OS and YT *vs*. YS) (Table 1).

### mRNA levels of adipogenic factors in response to aging and exercise in SOL muscle

**Figure 3:**
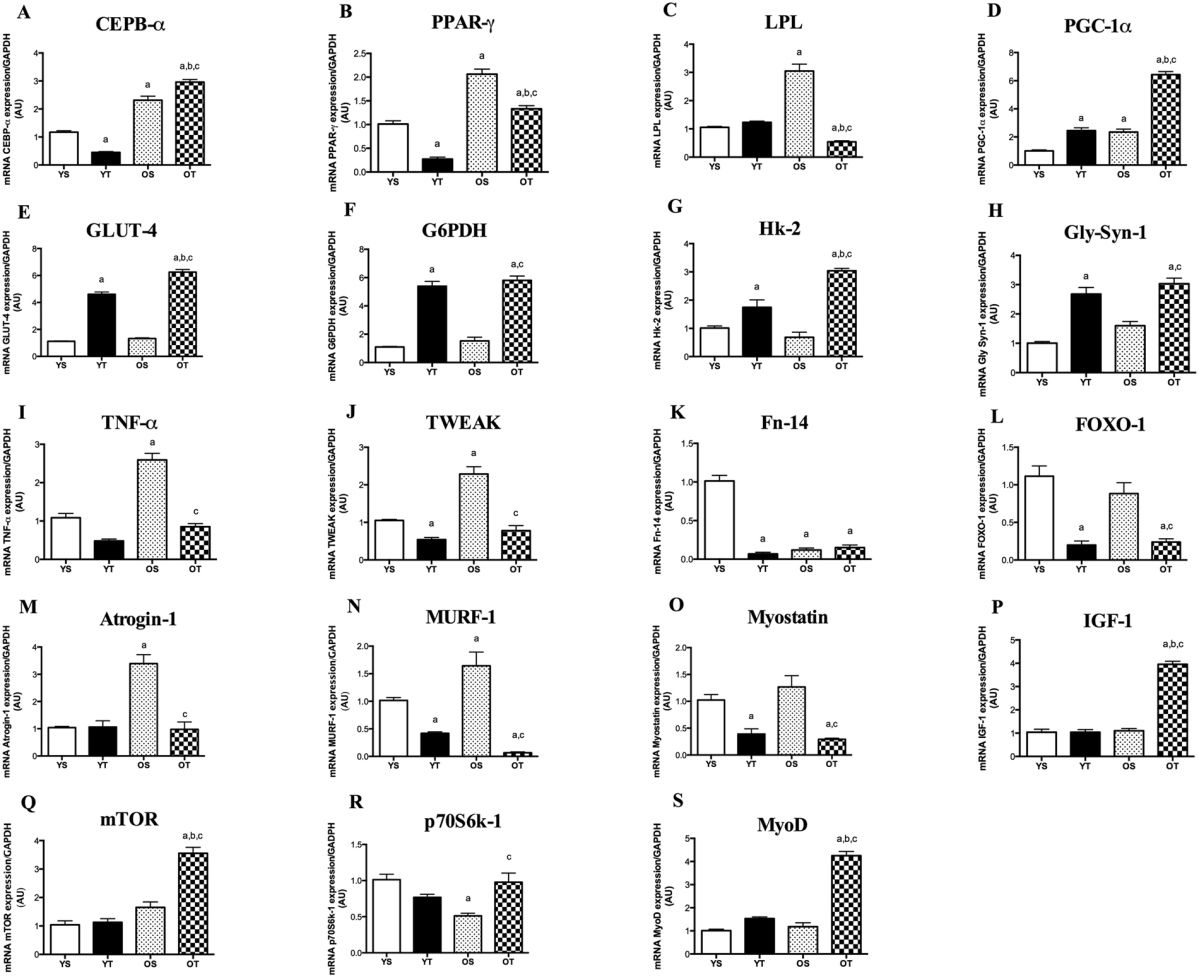
mRNA levels of CEBP-α (**A**), LPL (**B**), PPAR-γ (**C**), PGC-1α (**D**), Gly-Syn-1 (**E**), GP6DH (**F**), GLUT-4 (**G**) and Hk-2 (**H**) TNF-α (**I**), TWEAK (**J**), Fn-14 (**K**) FOXO-1 (**L**), Atrogin-1 (**M**), MURF-1 (**N**) Myostatin (**O**), IGF-1 (**P**), mTOR (**Q**), p70S6k-1 (**R**) and MyoD (**S**) of SOL muscle. Groups: young sedentary (YS), young trained (YT), old sedentary (OS) and old trained (OT). Values are expressed as means ± SEM. Two-way ANOVA, p < 0.05: ^a^vs. YS; ^b^vs. YT; ^c^vs. OS. n = 6/group.

CEBP-α, LPL, PPAR-γ and PGC-1α mRNA levels were elevated with aging (Fig. 3A–D). In response to exercise training, RT elevated mRNA levels of CEBP-α and PGC-1α in SOL muscle (Fig. 3A and D), whereas PPAR-γ and LPL were reduced in OT in relation to OS animals (Fig. 3B and C). In young rats, RT led to decreases in CEBP-α and PPAR-y mRNA levels, even though PGC-1α was increased in YT rats in relation to their age-matched counterparts.

### mRNA levels of glucose metabolism regulators of SOL muscle in response to aging and exercise

Aging did not affect GLUT-4, G6PDH, Hk-2 and Gly-synt-1 mRNA levels in SOL muscle (Fig. 3E–H). However, all those transcripts were elevated in trained rats when compared with their matched sedentary group (OT *vs*. OS and OT *vs*. YS).

### mRNA levels of atrophy and hypertrophy-related factors of SOL muscle in response to aging and resistance training

RT was effective at mitigating the age-associated increase of TNF-α, TWEAK, Atrogin-1 and MURF-1 mRNA levels in SOL muscle (OT *vs*. OS) (Fig. 3I,J,M and N). Likewise, RT decreased FOXO-1 and myostatin in OT rats when compared with OS and YS animals (Fig. 3L and O). Moreover, TWEAK, Fn-14, FOXO-1, MURF-1, and myostatin mRNA levels were reduced after RT in young rats (YT *vs*. YS) (Fig. 3J,K,L,N and O). Interestingly, Fn-14 transcript was lower in OS and OT rats in relation to YS (Fig. 3K). On the other hand, IGF-1, mTOR and MyoD mRNA levels were not affected by aging (Fig. 3P,Q and S), even though p70S6k-1 transcript was significantly decreased in OS rats when compared with YS (Fig. 3R). Conversely, each of those transcripts was substantially elevated in OT animals in comparison with OS rats (Fig. 3P–S).

### mRNA levels of adipogenic factors in response to aging and exercise in GAS muscle

**Figure 4:**
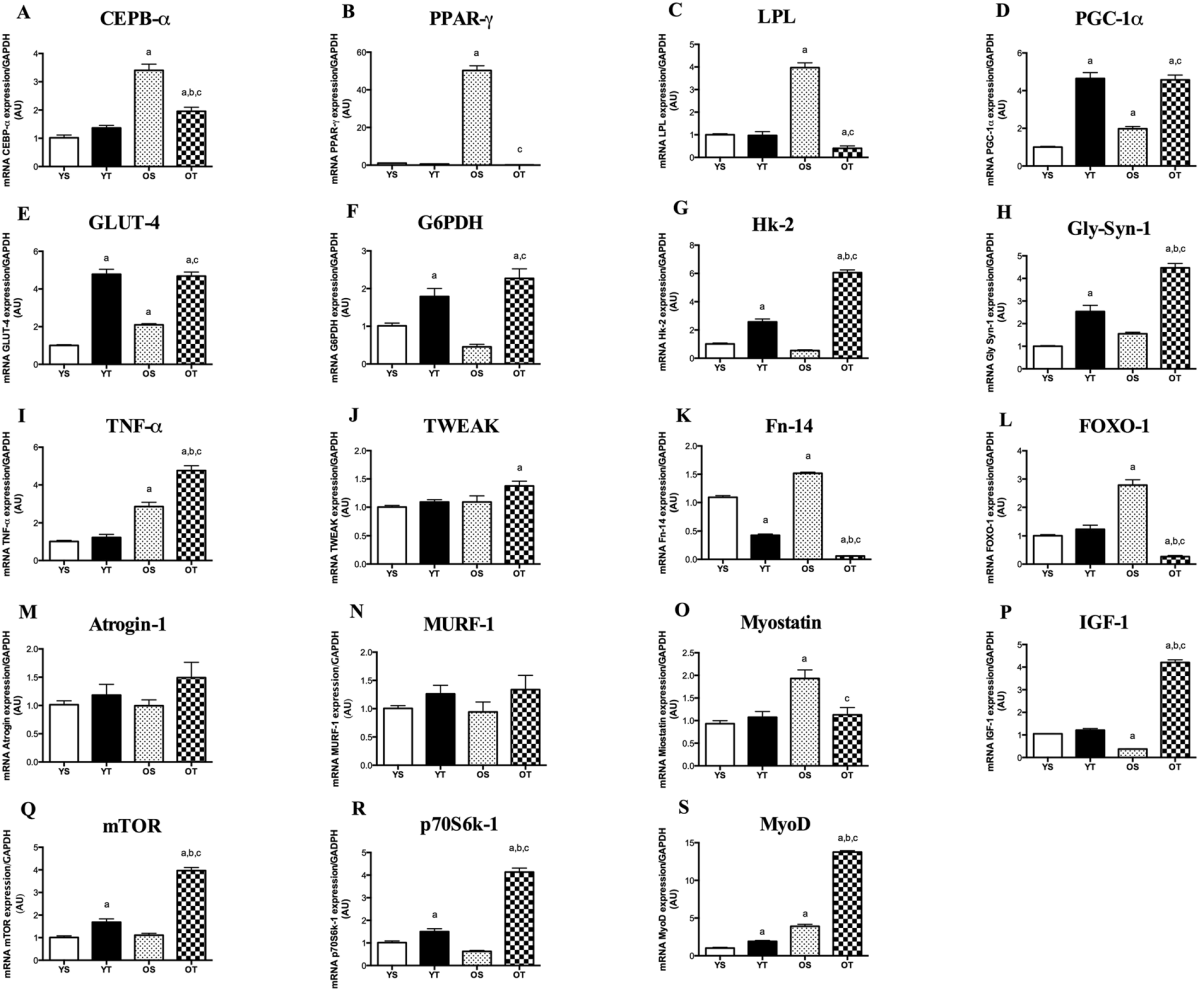
mRNA levels of CEBP-α (**A**), LPL (**B**), PPAR-γ (**C**), PGC-1α (**D**), Gly-Syn-1 (**E**), GP6DH (**F**), GLUT-4 (**G**) and Hk-2 (**H**) TNF-α (**I**), TWEAK (**J**), Fn-14 (**K**) FOXO-1 (**L**), Atrogin-1 (**M**), MURF-1 (**N**) Myostatin (**O**), IGF-1 (**P**), mTOR (**Q**), p70S6k-1 (**R**) and MyoD (**S**) of GAS muscle. Groups: young sedentary (YS), young trained (YT), old sedentary (OS) and old trained (OT). Values are expressed as means ± SEM. Two-way ANOVA, p < 0.05: ^a^vs. YS; ^b^vs. YT; ^c^vs. OS. n = 6/group.

RT alleviated the age-induced increases of CEBP-α, PPAR-γ and LPL transcripts in GAS muscle, although mRNA levels of PGC-1α increased in OT in relation to OS rats (Fig. 4A–D). PGC-1α transcript was also elevated in YT rats when compared with YS (Fig. 4D).

### mRNA levels of glucose metabolism regulators of GAS muscle in response to aging and exercise

GLUT-4 transcript was found to be greater in OS than in YS rats in GAS muscle (Fig. 4E). Similar to SOL, GLUT-4, G6PDH, Hk-2 and Gly-synt-1 mRNA levels increased in GAS muscle of trained rats when compared with their matched sedentary group (OT *vs*. OS and OT *vs*. YS) (Fig. 4E–H).

### mRNA levels of atrophy and hypertrophy-related factors of GAS muscle in response to aging and resistance training

**Figure 5:**
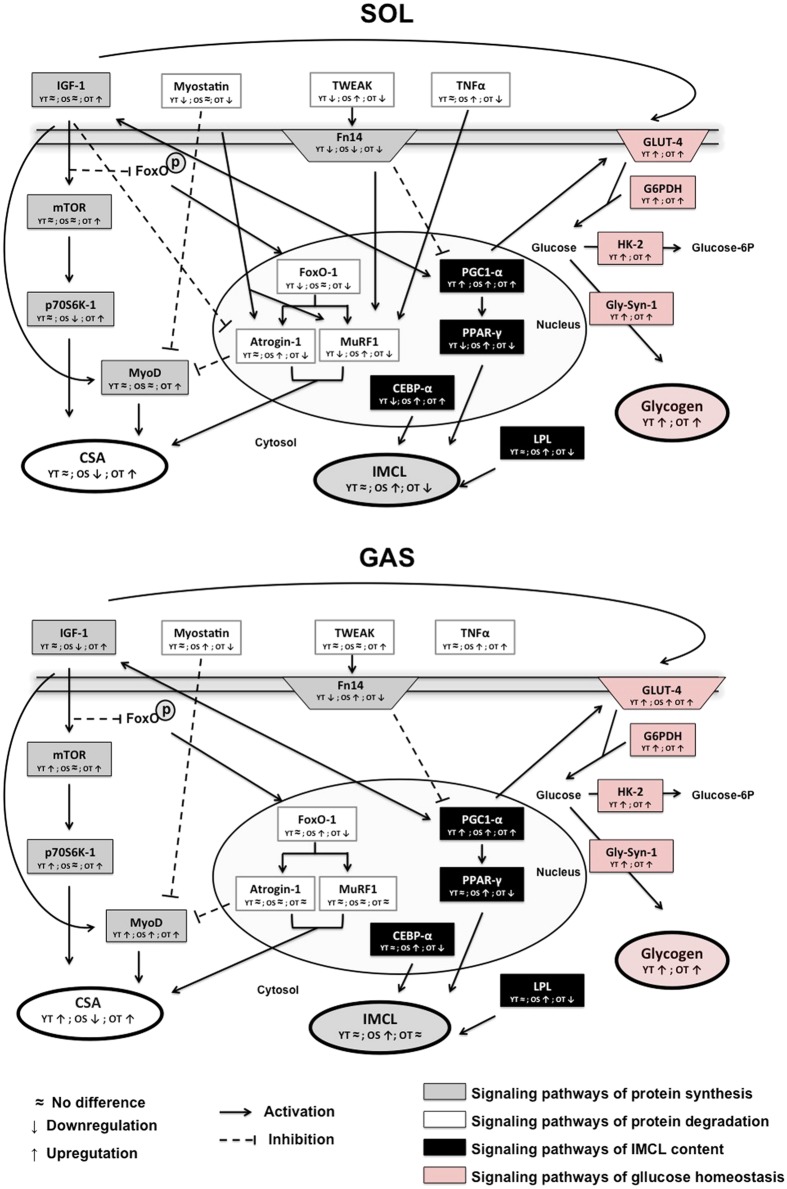
Integrative intracellular signaling of muscle atrophy/hypertrophy, IMCL accumulation and glycogen content in SOL and GAS muscles. IGF-1 has pleiotropic functions, some of which could be attributed to activation of satellite cells, as indicated by MyoD expression, and inhibition of ubiquitin ligases (Atrogin-1 and MURF-1) by FOXO1/3 mechanism. Myostatin seems to control MyoD levels in both muscles. Age-induced muscle atrophy and IMCL accumulation seem to be triggered by interplay between IGF-1 and PGC-1α as well as glucose uptake oxidation and storage through GLUT-4 and G6PDH. PGC-1α was inhibited by TWEAK/Fn-14 signaling in both muscles while TNF-α seems to modulate PGC-1α only in SOL muscle. LPL might be modulating IMCL content, although we demonstrated increases both in mRNA levels of LPL and IMCL in OS rats.

TNF-α, Fn-14, FOXO-1 and Myostatin mRNA levels were significantly greater in GAS muscle of OS compared with YS rats (Figs 4I,K and 5E). No changes were observed in mRNA levels of MURF-1 and Atrogin-1 among the groups (Fig. 4M,N). Whereas RT further elevated the age-induced increases of TNF-α mRNA levels (Fig. 4I), Fn-14, FOXO-1 and Myostatin transcripts were reduced in GAS muscle of OT rats when compared with OS rats (Fig. 4K,L and O). While TWEAK mRNA levels were increased, Fn-14 transcript was significantly reduced in OT rats (*vs*. OS) (Fig. 4J,K). RT also reduced FN-14 transcript in young rats (YT *vs*. YS). As expected, based on the evidence from atrophic markers, RT in aged rats resulted in marked increases of IGF-1, mTOR, p70S6k-1 and MyoD transcripts in GAS muscle (OT *vs*. OS) (Fig. 4P–S). However, while aging evoked decreases in mRNA levels of IGF-1 (Fig. 4P), MyoD transcript was elevated in OS rats when compared with YS group (OS *vs*. YS) (Fig. 4S). In young rats, RT was able to increase mTOR, p70S6k-1 and MyoD mRNA levels in relation to YS group (Fig. 4Q–S).

## Discussion

To our knowledge, we have demonstrated for the first time that RT lowers IMCL content, which was associated with downregulation of PPAR-y gene expression in skeletal muscle. In addition, ours results indicate that PGC-1α seems to transcriptionally mediate intracellular signaling related to age-dependent changes in glucose homeostasis and fiber size in skeletal muscle. Accordingly, these responses were related to decreased expression of key catabolic genes and increased expression of key anabolic genes. Indeed, our findings contribute to a better understanding of sarcopenia and its relation with skeletal muscle metabolism during aging. Our findings demonstrate the effect of RT in aged muscle is an effective physiological intervention capable of counteracting lipid accumulation within the muscle, and thus, sarcopenia.

In fact, IMCL accumulation has been reported with advancing age^3^, which supports our findings. Although endurance exercise training led to an increase in IMCL content in the elderly^23^, the effects of RT are unknown. Here, we demonstrated that RT decreased the age-induced IMCL accumulation in SOL, but not in GAS, suggesting that RT plays a distinct role on IMCL content that likely depends on fiber composition of the muscle^24^. Concomitant to age-induced increases in IMCL content, elevated adipogenic markers were observed in GAS or SOL muscles of old rats, suggesting that aging affects the early stages of cell signaling of adipocyte differentiation and lipoprotein metabolism in skeletal muscle. PPARγ and C/EBP-α are key adipogenic transcription factors^6^ that regulate the expression of genes related to lipogenesis in skeletal muscle^7^. PPARγ mRNA expression has been positively correlated with triglyceride concentration in skeletal muscle^7^. Indeed, we demonstrated increased mRNA levels of PPARγ with aging (OS *vs*. YS) in both muscles. Our findings suggest that age-induced IMCL accumulation could be associated with elevated PPARγ and C/EBP-α mRNAs expressions. However, mRNA levels of PPARγ has been shown to decrease with aging in skeletal muscle of old rats^25^.

In response to exercise training, we observed lower PPARγ transcripts in SOL and GAS muscles of OT rats (*vs*. OS), suggesting RT might play a critical role in reducing IMCL, which likely depends on fiber composition of the muscle^24^, given that IMCL was not diminished in GAS of OT rats. Contrary to our findings, exercise training has been shown to increase PPARγ protein expression in EDL muscle of rats^26^ and mRNA levels in soleus and plantaris muscle of rats^27^. While C/EBP-α transcripts increased both in SOL and GAS with advancing age, the effects of RT were distinct between the muscles in old rats. Thus, we hypothesize that C/EBPα would not be involved in the reduction of IMCL content induced by RT in old rats. Therefore, decreases of IMCL content induced by RT could be linked to lower mRNA levels of PPARγ, but not for C/EBPα transcripts.

Control of intramuscular triglyceride metabolism involves LPL activity^28^. Interestingly, we observed increased LPL synthesis in both muscles, although LPL protein content and activity decreases in SOL muscle in old rats^29^. Conversely, RT led to a marked reduction in LPL transcripts in old rats. However, LPL activity increases after exercise training^30^. Other have shown that LPL mRNA levels were elevated after running training in rats^31^. Thus, we postulate that LPL regulation in skeletal muscle seems to be regulated at either a post-transcriptional/pretranslational level (and not by LPL synthesis).

The PPARy co-activator, PGC-1α has been shown to be a potent transcriptional factor that regulates lipid metabolism^32^ and glycogen content^33^. A previous study found reduction in PGC-1α expression with aging^34^. Importantly, overexpression of PGC-1α in the muscle protects against development of sarcopenia in old mice^35^. However, we found increased mRNA levels of PGC-1α with aging, even though it was not enough to prevent the reduction of CSA in GAS and SOL muscles in OS rats.

Indeed, PGC-1α has been shown to consistently increase in skeletal muscle after exercise training^36,37^, whereas high intensity training elicited increased content of nuclear PGC-1α, while no changes in protein content was observed^38^. In this regard, PGC-1α in combination with exercise training improves glucose homeostasis in mice^39^. Rather than protein content, our findings demonstrate increased PGC-1α mRNA levels after RT, suggesting that mitochondrial biogenesis might be triggered by high intensity RT at the transcriptional level. Accordingly, while RT attenuated the age-associated reductions in CSA, elevated PGC-1α transcript was observed in SOL and GAS muscles of OT rats, suggesting that PGC-1α might be involved in protective mechanisms of RT in order to prevent muscle wasting^37^.

Aging did not affect glycogen content, Gly-Syn-1, G6PDH, Hk-2 and GLUT-4 mRNA levels, either in GAS or SOL muscles. Similarly, glycogen content was not altered in aged SOL muscle^40^, while other observed a reduction in glycogen content in soleus of old rats^41^. In contrast, RT evoked greater glycogen content, which could be related with upregulation of GLUT-4, G6PDH, Hk-2 and Gly-Syn-1 transcripts observed in this study. These data suggest that RT plays a large role in glycogen synthesis, glucose uptake, transport and metabolism, even at a transcriptional level.

Indeed, GLUT-4, G6PDH and Hk-2 are determinants of glucose uptake within skeletal muscle during exercise^42^. Increased GLUT-4 has been consistently documented after exercise training^42^. In fact, endurance training induced greater glycogen concentration, which was associated with elevated protein abundance of GLUT-4, Hk-2 and glycogen synthase in older people^43^. In this context, glucose-6-phosphate dehydrogenase (G6PDH) was recently associated with glucose uptake^44^, although this enzyme has been poorly studied in skeletal muscle^20^. Whereas aging did not affect G6PDH mRNA levels in this study, its activity and protein content was reduced in the gastrocnemius of aged rodents^45^.

Evidence is scarce concerning the effects of physical training on G6PDH levels. However, it was shown that endurance training reduced G6PDH in adipose tissue of mice^46^ while our findings revealed that RT increased mRNA levels in G6PDH in both skeletal muscles tested. Given that glucose is transported into muscle cells, hexokinase plays an essential role in its conversion to glucose 6-phosphate and synthesis of glycogen, since glycogen synthase is activated by glucose 6-phosphate^47^. Considering hexokinase participates of glucose transport and uptake and hexokinase II is the most predominant isoform in rat skeletal muscle^48^, we evaluated Hk-2 in GAS and SOL muscles. Whereas no changes were found in Hk-2 transcript with advancing age in this study, reduction in Hk-2 mRNA levels was observed^49^. Conversely, RT elevated Hk-2 mRNA expression, either in GAS or SOL muscles, which is in partial agreement with other. For instance, increased Hk-2 protein levels has been observed following running sessions in mice^50^.

Although our results demonstrated no changes in Gly-Syn-1 transcripts with aging, decreases in Gly-Syn activity and protein levels have been reported in skeletal muscle of old rats^40,41^. It has been reported that chronic exercise training leads to an increase in both Gly-Syn activity and protein expression in rats^51^. Taken together, as similar effects of RT were observed in GLUT-4, G6PDH, Hk-2 and Gly-Syn-1, we suggest that RT plays a crucial role in glucose homeostasis (uptake, transport and metabolism) at a transcriptional level along with upregulation of PGC-1α in aged skeletal muscle after RT. Furthermore, PGC-1α controls GLUT-4 gene expression, suggesting its importance on exercise training-mediated glucose uptake^36^. Considering PGC-1α transcripts were dramatically elevated in GAS muscle of YT rat, we suggest distinct effects of exercise training on PGC-1α signaling between skeletal muscle and aging.

It’s noteworthy that PGC-1α is considered a fundamental intracellular target because of its importance in atrophy signaling^37^. This is supported, in part, by studies demonstrating that PGC-1α was inhibited by TWEAK/Fn14 signaling^52^ while PGC-1α activates the IGF-1-mTOR pathway^53^. In fact, our findings demonstrated increases in PGC-1α mRNA coincided with augmented IGF-1/mTOR/p70S6k-1 transcripts, both in SOL and GAS muscles of OT rats. Increases in age-induced TNF-α, TWEAK, Atrogin-1 and MURF-1 mRNA levels were observed in SOL, while Fn-14, FOXO-1 were unchanged. These responses were accompanied by a reduction in fiber CSA of SOL muscle from OS rats. Inflammatory cytokines, such as TNF-α, initiate downstream signaling involved in muscle atrophy^54^. In this context, TWEAK-Fn14 system has emerged as a regulator of muscle atrophy, mitochondrial dysfunction and slow-to-fast fiber type switching^13^. In fact, as TWEAK was elevated in SOL muscle, we observed concomitant increases in Atrogin-1 and MURF-1 transcripts. However, while no changes were noted in MURF-1 and Atrogin-1 transcripts in GAS muscle with aging in this study, Atrogin-1 and MURF1 were downregulated in skeletal muscle of old rats^55^, as other have shown elevations in mRNA levels of these E3 ligases^14^. Given the inconsistent findings, we suggest that regulation of Atrogin-1 and MURF-1 seems to occur in a fiber-type specific manner in aged skeletal muscles^24^.

In GAS muscle, elevated transcripts of TNF-α, Fn-14, FOXO-1 and myostatin were observed with aging, whereas no changes were observed in TWEAK, Atrogin-1 and MURF-1. The unchanged Atrogin-1 and MURF-1 transcripts observed in aged GAS muscle might not be contributing to the decreases in CSA and sarcopenia, as suggested by others^56^. Myostatin, a negative regulator of muscle growth, and shown to increase with aging^57,58^, is also associated with upregulation of MURF-1 and Atrogin-1^59^. Moreover, myostatin-induced upregulation of Atrogin-1 is mediated by FOXO-1^15^. However, we did not demonstrate a similar fashion of crosstalk between signaling pathways. These discrepancies could be due to our findings solely rely on transcriptional mechanisms. Thus, aging might modulate atrophic signaling pathways, not only at a post-translational mechanism but also at transcriptional control.

Age-stimulated increases in mRNA of TNF-α, TWEAK, Atrogin-1 and MURF-1 were blunted in SOL muscle after RT. On the other hand, RT reduced the age-induced increases of Fn-14, FOXO-1 in GAS muscle. Myostatin mRNA expression was significantly decreased after RT, in agreement with the finding from previous report^58^. Indeed, RT attenuated the age-associated elevations of TNF-α in vastus lateralis from elderly humans^60^. Furthermore, recent evidence has shown that TWEAK-Fn14 transcripts are responsive to RT^61^. Similarly, others reported decreases in MURF-1 protein content after endurance training^57^. To our knowledge, this is the first evidence that RT downregulated the major intracellular regulators of age-induced atrophy of SOL and GAS muscles. Therefore, we propose that RT modulates transcription in aged muscles in order to minimize the age-associated atrophic signaling.

RT has been shown to alleviate the age-induced reduction in CSA in GAS and SOL muscles, which is likely due in part to pro-growth signaling events that result in increased rates of protein synthesis^62^. In fact, IGF-1, mTOR, p70S6k-1 and MyoD were markedly upregulated after RT, either in both GAS and SOL muscles of old rats. These findings are in support of the hypothesis that RT modulates the hypertrophic signaling, even at transcriptional mechanisms. p70S6k-1 transcript was elevated in OT rats, suggesting RT plays crucial role on downstream hypertrophy signaling, which are supported by other^63^. Our findings corroborate with other, once MyoD transcripts were elevated in vastus lateralis after RT in old women^58^. IGF-1 has pleiotropic functions, some of which could be attributed to activation of satellite cells, as indicated by MyoD expression^64^ and inhibition of ubiquitin ligases (Atrogin-1 and MURF-1)^65^. With advancing age, diminished activation of IGF-AKT-mTOR axis occurs, resulting in FOXO1/3-mediated activation of Atrogin-1 and leading to protein degradation^37^. Furthermore, IGF-1 favors free fatty acid uptake and oxidation in skeletal muscle^66^. Thus, these responses might be dependent on, at least in part, a complex interplay of PGC-1α, GLUT-4 and IGF-1. Accordingly, our findings suggest that RT plays a vital role at a transcriptional level on intramuscular metabolic metabolism and signaling related to myofiber size during aging, as proposed by the intracellular signaling. Considering that intracellular crosstalk between lipogenesis, glucose homeostasis and muscle development might be occurring in response to aging and RT, we have proposed an outline of the pathways, including events occurring at the transcriptional level (Fig. 5).

Finally, it is important to point out some limitations of the present study. Although we evaluated regulators of intramuscular lipogenesis and glucose homeostasis, along with muscle atrophy and hypertrophy pathways, we did not measure protein content and subcellular localization. In addition, we evaluated RPLP0 expression, which was found to vary according in the muscles and groups (data not shown). Thus, RPLP0 might not be a good housekeeping gene for GAS and SOL muscles in a model of aging and RT in mice.

## Conclusions

In conclusion, RT attenuated the age-associated accumulation of IMCL concomitant to a downregulation of PPARγ gene expression and enhanced expression of glucose homeostasis regulators (GLUT-4, G6PDH, Hk-2 and Gly-Syn-1). These responses were also linked to decreasing catabolic (TNF-α, TWEAK/Fn14 axis; FOXO-1, Atrogin-1 and MURF1; myostatin) and increasing anabolic (IGF-1-mTOR-p70S6sk-1 axis; MyoD) signaling effectors. Our results point out the importance of RT on modulation of gene expression of intracellular regulators related to age-related morphological and metabolic adaptations of skeletal muscle.

## Methods

Twenty-eight male Wistar rats with 3 (n = 14; 298.74 ± 32 g) and 20 months old (n = 14; 517.8 ± 76 g) of age were housed in plastic cages under controlled environmental conditions (12-hour light/dark cycle) with free access to water and standard chow (Socil, São Paulo, Brazil). Rats were randomly distributed into four experimental groups with 6 animals per group in the following order: young sedentary (YS), young trained (YT), old sedentary (OS) and old trained (OT). The experimental procedures received approval from the Animal Experimentation Ethics Committee of the Federal University of São Carlos, SP, Brazil (number 056/2010), and the study was conducted in accordance with the National Guide for the Care and Use of Laboratory Animals.

### Resistance Training (RT) protocol

The description of RT protocol was recently reported by our laboratory^67^.

### Body Weight and Muscle Sample Collection

Initial (iBW) and final (fBW) body weights were recorded in grams (g) before the first session of RT protocol and 48 hours after the last training session, respectively. Forty-eight hours after the last training session, animals were anesthetized using i.p injection with a solution of xylazine (12 mg/Kg of BW) and euthanized using ketamine (95 mg/Kg of body weight). Soleus (SOL) and Gastrocnemius (GAS) muscles were carefully removed and weighed. The muscles were then divided into two parts at the middle of the belly: the proximal (origin) and the distal (insertion) attachments were used for the histological analysis and mRNA analysis, respectively. For histological evaluation, the muscle fragment was immediately frozen in isopentane, pre-cooled in liquid nitrogen and stored at −80 °C (Forma Scientific, Marietta, Ohio). For mRNA analysis, the muscle fragment was frozen in liquid nitrogen and stored at −80 °C.

### Histological Analysis

Histological cross-sections (10 µm) of each GAS and SOL muscles were obtained in a cryostat (Micron HE 505, Jena, Germany). IMCL content was determined using Oil Red staining and quantified as previously described^68^. Ten-µm slices were stained with Toluidine Blue/1% Borax (TB) in order to measure fiber cross-sectional area (CSA). Images of 5 different regions were obtained using a light microscope (Axiolab, Carl Zeiss, Jena, Germany) equipped with a digital camera (Sony DSC S75, Tokyo, Japan). The CSA of 100 randomly fibers were chosen from each picture and measured using the Axiovision 3.0.6 SP4 software (Carl Zeiss, Jena, Germany) totaling 600 muscle fibers per animal. Finally, CSA of muscle fibers were normalized by fBW of matched animal (CSA/body weight).

### Glycogen content

SOL and GAS muscle samples were processed with hot 30% KOH and glycogen was precipitated by ethanol to determine muscle glycogen as previously described^69^.

### RNA Isolation - PCR

Frozen fragment of each muscle was homogenized (Omni Tip Plastic Homogenizer Probes^®^ Kennesaw, GA, USA) and total RNA was isolated using Trizol reagent (Life Technologies). In order to obtain clean RNA with no contamination, samples were treated with DNase followed by removal treatment (Life Technologies). The amount of RNA was quantified by Qubit^®^ (Life Technologies) using 1 µL of each sample. The integrity and quality of the total RNA obtained was tested in a Bioanalyser (Agilent Technologies Inc. USA). The RIN (RNA Integrity Number) value ranged from 8.0 to 10.0, and the ratio ranged from 1.8 to 2.0. This indicated that intact RNA, free of genomic DNA, was successfully isolated. Approximately 1 µg of total RNA from each sample was used to synthesize cDNA using the High Capacity cDNA Reverse Transcription Kit (Applied Biosystems) according to the manufacturer’s instructions.

### Real Time-PCR

The differential expression of genes was validated by qPCR using Sybr Green PCR Master Mix (Life Technology^®^). Amplifications were performed by qPCR using 10–80 ng cDNA/µl added to a reaction containing 10 μM SYBR Green PCR master mix and 100–300 nM primers (sense and antisense) in a final volume of 30 µl solution in triplicate. The cycling conditions were in accordance to the standards of each primer according to the annealing temperature. The Cq value (Cycle Quantification) of each sample was calculated using the StepOne software (Applied Biosystems). The average Cq values of triplicates were used for the calculation of the Fold Change (Arbitrary Unit). Six animals from each experimental group were used for Real Time-PCR analysis (n = 6/group). GAPDH were used as reference genes for normalization. The gene expression assays were 100% efficient, with a slope value of −3.32 and r-value >0.99.

### Primers

Oligonucleotides for muscle-specific transcriptional factor were based on the work described by Hill and Goldspink^70^. The primers were constructed using the Primer Express software (Applied Biosystems, Foster City, CA) described in Table 2.

**Table 2:** List of oligonucleotides primers.

	Forward	Reverse	NCBI (Reference Sequence)	Amplicon Size, bp
CEBP-α	TCGGTGGATAAGAACAGCAA	GTTGCGCTGTTTGGCTTTA	NM_001287577.1	93
LPL	CTTCTTGATTTACACGGAGGT	ATGGCATTTCACAAACACTG	NM_012598.2	229
PPAR-y	AAGGGGCCTGGACCTCTGCTG	ATAAGGCGGGGACGCAGGCT	XM_006504151.1	630
PGC-1α	TCATGGAGCAATAAAGCGAAG	TGTGGGTTTGGTGTGAGGAG	XM_006503779.1	117
Gly-Synt-1	CCGGCTTTGGCTGCTTTAT	CCGATCCAGAATGTAAATGCC	NM_030678.3	71
GP6DH	GTTTGGCAGCGGCAACTAA	GGCATCACCCTGGTACAACTC	NM_017006.2	108
GLUT-4	CAAAGCATCGACCAGTGCTA	TGGACAGCACTGACTTCCAG	XM_006506283.1	190
Hk-2	ACCAAGTGCAGAAGGTTGACCA	TCTGGTGGCAGGGGAACGAGAA	NM_013820.3	432
TNFα	GCCACCACGCTCTTCTGTCT	GTCTGGGCCATGGAACTGAT	NM_012675.3	101
TWEAK	GCTACGACCGCCAGATTGGG	GCCAGCACACCGTTCACCAG	NM_011614.3	130
Fn14	AAGTGCATGGACTGCGCTTCTT	GGAAACTAGAAACCAGCGCCAA	NM_181086.3	154
FoxO1	TCAAGGATAAGGGCGACAGC	GTTCCTTCATTCTGCACTCGAAT	NM_019739.3	103
Atrogin-1	CCATCAGGAGAAGTGGATCTATGTT	GCTTCCCCCAAAGTGCAGTA	NM_133521.1	75
MuRF1	TGTCTGGAGGTCGTTTCCG	ATGCCGGTCCATGATCACTT	NM_080903.1	59
IGF-1	GCTCTTCAGTTCGTGTGTGGA	AGATCACAGCTCCGGAAGCA	NM_184052.3	125
mTOR	CACCCAAGCCTGGGACCTCTA	GGCTGGTTGGGGTCATATGTT	NM_019906.1	156
p70S6K-1	CTACAGAGACCTGAAGCCGGAGA	AATGTGTGCGTGACTGTTCCATC	NM_031985.1	114
Myostatin	CTACCACGGAAACAATCATTACCA	AGCAACATTTGGGCTTTCCAT	NM_019151.1	78
MyoD	ACTACAGCGGCGACTCAGAC	ACTGTAGTAGGCGGCGTCGT	NM_176079.1	122
RPLP0	AGGGTCCTGGCTTTGTCTGTGG	AGCTGCAGGAGCAGCAGTGG	NM_022402.2	135
GAPDH	TGCACCACCAACTGCTTA	GGATGCAGGGATGATGTTC	NM_017008.4	177

CEBP-α: CCAAT/enhancer binding proteins alpha; LPL: Lipoprotein lipase; PPAR-γ: Peroxisome proliferator activated receptor gamma; PGC-1α: Peroxisome proliferator-activated receptor gamma coactivator 1-alpha; Gly-Synt-1: Glycogen synthase-1; G6PDH: Glucose-6-phosphate dehydrogenase; GLUT-4: Glucose transporter type 4; Hk-2: Hexokinase-2; TNF-α: Tumor necrosis factor alpha; TWEAK: Tumor necrosis factor apoptosis inducing; Fn14: Growth factor-inducible 14 receiver fibroblasts; FoxO1: Forkhead box protein O1; Atrogin-1: F-box protein 32; MuRF1: Muscle ring finger protein-1; IGF-1: Insulin-like growth factor 1; mTOR: Mamalian target of rapamycin; p70S6K: p70S6 kinase 1; Myostatin: Growth differentiation factor 8; MyoD: myogenic growth factor 1 RPLP0: Ribosomal protein lateral stalk subunit P0; GAPDH: Glyceraldehyde-3-phosphate dehydrogenase.

### Data standardization

We measured GAPDH and RPLP0 as internal controls. However, we observed a high variability of RPLP0 mRNA than GAPDH. Therefore, the GAPDH gene was chosen as the internal control, assuming that the GAPDH mRNA was expressed constitutively^71^.

### Statistical Analysis

Results were expressed as means ± SEM. Shapiro-Wilk and Levene’s tests were used to investigate whether the data were normally distributed. As all included variables were normally distributed, a two-way ANOVA (training × age) followed by a Tukey HSD post-hoc test were performed to compare treatments. Differences were considered significant when p < 0.05. Statistical analysis was performed using the Statistica 7.0 software package (StatSoft Inc., Tulsa, OK, USA).

## References

[1] Walston JD (2012). Sarcopenia in older adults. Curr Opin Rheumatol.

[2] Lushaj EB, Johnson JK, McKenzie D, Aiken JM (2008). Sarcopenia accelerates at advanced ages in Fisher 344xBrown Norway rats. J. Gerontol. A. Biol. Sci. Med. Sci..

[3] Crane JD, Devries MC, Safdar A, Hamadeh MJ (2010). & Tarnopolsky, M. a. The effect of aging on human skeletal muscle mitochondrial and intramyocellular lipid ultrastructure. J. Gerontol. A. Biol. Sci. Med. Sci..

[4] Shulman GI (2000). Cellular mechanisms of insulin resistance. Journal of Clinical Investigation.

[5] Højlund K, Beck-Nielsen H (2006). Impaired glycogen synthase activity and mitochondrial dysfunction in skeletal muscle: markers or mediators of insulin resistance in type 2 diabetes?. Curr. Diabetes Rev..

[6] Moseti, D., Regassa, A. & Kim, W. K. Molecular regulation of adipogenesis and potential anti-adipogenic bioactive molecules. *International Journal of Molecular Sciences* **17** (2016). 10.3390/ijms17010124 PMC4730365 26797605

[7] Kannisto K (2006). Differential expression of peroxisomal proliferator activated receptors alpha and delta in skeletal muscle in response to changes in diet and exercise. Int. J. Mol. Med..

[8] Vega RB, Huss JM, Kelly DP (2000). The coactivator PGC-1 cooperates with peroxisome proliferator-activated receptor alpha in transcriptional control of nuclear genes encoding mitochondrial fatty acid oxidation enzymes. Mol. Cell. Biol..

[9] Puigserver P, Spiegelman BM (2003). Peroxisome proliferator-activated receptor-gamma coactivator 1 alpha (PGC-1 alpha): transcriptional coactivator and metabolic regulator. Endocr. Rev..

[10] Summermatter S, Troxler H, Santos G, Handschin C (2011). Coordinated balancing of muscle oxidative metabolism through PGC-1α increases metabolic flexibility and preserves insulin sensitivity. Biochem. Biophys. Res. Commun..

[11] Goldberg IJ (1996). Lipoprotein lipase and lipolysis: central roles in lipoprotein metabolism and atherogenesis. J. Lipid Res..

[12] Houtkooper RH (2011). The metabolic footprint of aging in mice. Sci. Rep..

[13] Sato, S., Ogura, Y. & Kumar, A. TWEAK/Fn14 signaling axis mediates skeletal muscle atrophy and metabolic dysfunction. *Frontiers in Immunology* **5** (2014). 10.3389/fimmu.2014.00018 PMC3902304 24478779

[14] Clavel S (2006). Atrophy-related ubiquitin ligases, atrogin-1 and MuRF1 are up-regulated in aged rat Tibialis Anterior muscle. Mech. Ageing Dev..

[15] Lokireddy S (2011). Myostatin induces degradation of sarcomeric proteins through a Smad3 signaling mechanism during skeletal muscle wasting. Mol. Endocrinol..

[16] Breen L, Phillips SM (2011). Skeletal muscle protein metabolism in the elderly: Interventions to counteract the ‘anabolic resistance’ of ageing. Nutr. Metab. (Lond)..

[17] Bonaldo P, Sandri M (2013). Cellular and molecular mechanisms of muscle atrophy. Dis. Model. Mech..

[18] Yin H, Price F, Rudnicki MA (2013). Satellite cells and the muscle stem cell niche. Physiol. Rev..

[19] Hunter GR, McCarthy JP, Bamman MM (2004). Effects of resistance training on older adults. Sports Med..

[20] Brioche T, Pagano AF, Py G, Chopard A (2016). Muscle wasting and aging: Experimental models, fatty infiltrations, and prevention. Molecular Aspects of Medicine.

[21] Knuiman P, Hopman MTE, Mensink M (2015). Glycogen availability and skeletal muscle adaptations with endurance and resistance exercise. Nutr. Metab. (Lond)..

[22] Devries MC (2013). Endurance training modulates intramyocellular lipid compartmentalization and morphology in skeletal muscle of lean and obese women. J. Clin. Endocrinol. Metab..

[23] Pruchnic R (2004). Exercise training increases intramyocellular lipid and oxidative capacity in older adults. Am J Physiol Endocrinol Metab.

[24] Delp MD, Duan C (1996). Composition and size of type I, IIA, IID/X, and IIB fibers and citrate synthase activity of rat muscle. J. Appl. Physiol..

[25] Ye P, Zhang X-J, Wang Z-J, Zhang C (2006). Effect of aging on the expression of peroxisome proliferator-activated receptor gamma and the possible relation to insulin resistance. Gerontology.

[26] Kawamura T (2004). Regulation of skeletal muscle peroxisome proliferator-activated receptor gamma expression by exercise and angiotensin-converting enzyme inhibition in fructose-fed hypertensive rats. Hypertens. Res..

[27] Spangenburg EE, Brown DA, Johnson MS, Moore RL (2009). Alterations in peroxisome proliferator-activated receptor mRNA expression in skeletal muscle after acute and repeated bouts of exercise. Mol. Cell. Biochem..

[28] Oscai LB, Essig DA, Palmer WK (1990). Lipase regulation of muscle triglyceride hydrolysis. J. Appl. Physiol..

[29] Bey L, Areiqat E (2001). Sano, a & Hamilton, M. T. Reduced lipoprotein lipase activity in postural skeletal muscle during aging. J. Appl. Physiol..

[30] Ong JM, Simsolo RB, Saghizadeh M, Goers JW, Kern PA (1995). Effects of exercise training and feeding on lipoprotein lipase gene expression in adipose tissue, heart, and skeletal muscle of the rat. Metabolism..

[31] Hamilton MT (1998). Role of local contractile activity and muscle fiber type on LPL regulation during exercise. Am. J. Physiol..

[32] Gouspillou, G. *et al*. The relationship between muscle fiber type-specific PGC-1α content and mitochondrial content varies between rodent models and humans. *PLoS One* **9** (2014). 10.1371/journal.pone.0103044 PMC4133187 25121500

[33] Wende AR (2007). A role for the transcriptional coactivator PGC-1alpha in muscle refueling. J. Biol. Chem..

[34] Kang C, Chung E, Diffee G, Ji LL (2013). Exercise training attenuates aging-associated mitochondrial dysfunction in rat skeletal muscle: Role of PGC-1α. Exp. Gerontol..

[35] Wenz T, Rossi SG, Rotundo RL, Spiegelman BM, Moraes CT (2009). Increased muscle PGC-1alpha expression protects from sarcopenia and metabolic disease during aging. Proc. Natl. Acad. Sci. USA.

[36] Baar K (2002). Adaptations of skeletal muscle to exercise: rapid increase in the transcriptional coactivator PGC-1. FASEB J..

[37] Ji LL, Kang C (2015). Role of PGC-1α in sarcopenia: Etiology and potential intervention - A mini-review. Gerontology.

[38] Little JP, Safdar A, Wilkin GP, Tarnopolsky MA, Gibala MJ (2010). A practical model of low-volume high-intensity interval training induces mitochondrial biogenesis in human skeletal muscle: potential mechanisms. J. Physiol..

[39] Summermatter S (2013). PGC-1α improves glucose homeostasis in skeletal muscle in an activity-dependent manner. Diabetes.

[40] Montori-Grau M (2009). Effects of aging and calorie restriction on rat skeletal muscle glycogen synthase and glycogen phosphorylase. Exp. Gerontol..

[41] Dall’Aglio E, Chang H, Reaven GM, Azhar S (1987). Age-related changes in rat muscle glycogen synthase activity. J Gerontol.

[42] Richter E (2013). a & Hargreaves, M. Exercise, GLUT4, and skeletal muscle glucose uptake. Physiol. Rev..

[43] Bienso, R. S. *et al*. Effects of Exercise Training on Regulation of Skeletal Muscle Glucose Metabolism in Elderly Men. *Journals Gerontol. Ser. A Biol. Sci. Med. Sci*. 1–7, 10.1093/gerona/glv012 (2015). 10.1093/gerona/glv012 25991826

[44] Lee-Young RS (2016). Glucose-6-phosphate dehydrogenase contributes to the regulation of glucose uptake in skeletal muscle. Mol. Metab..

[45] Kovacheva EL, Sinha Hikim AP, Shen R, Sinha I, Sinha-Hikim I (2010). Testosterone supplementation reverses sarcopenia in aging through regulation of myostatin, c-Jun NH2-terminal kinase, Notch, and Akt signaling pathways. Endocrinology.

[46] Higa TS, Spinola AV, Fonseca-Alaniz MH, Evangelista FS (2014). Remodeling of white adipose tissue metabolism by physical training prevents insulin resistance. Life Sci..

[47] Jensen J, Lai Y-C (2009). Regulation of muscle glycogen synthase phosphorylation and kinetic properties by insulin, exercise, adrenaline and role in insulin resistance. Arch. Physiol. Biochem..

[48] Lawrence GM, Trayer IP (1985). The localization of hexokinase isoenzymes in red and white skeletal muscles of the rat. Histochem. J..

[49] Tsao TS, Burcelin R, Charron MJ (1996). Regulation of hexokinase II gene expression by glucose flux in skeletal muscle. J. Biol. Chem..

[50] Röckl KSC (2007). Skeletal muscle adaptation to exercise training: AMP-activated protein kinase mediates muscle fiber type shift. Diabetes.

[51] Manabe Y (2013). Exercise training-induced adaptations associated with increases in skeletal muscle glycogen content. FEBS J..

[52] Hindi SM (2014). Regulatory circuitry of TWEAK-Fn14 system and PGC-1α in skeletal muscle atrophy program. FASEB J..

[53] Ruas JL (2012). A PGC-1α isoform induced by resistance training regulates skeletal muscle hypertrophy. Cell.

[54] Degens H (2010). The role of systemic inflammation in age-related muscle weakness and wasting: Review. Scandinavian Journal of Medicine and Science in Sports.

[55] Edström E, Altun M, Hägglund M, Ulfhake B (2006). Atrogin-1/MAFbx and MuRF1 are downregulated in aging-related loss of skeletal muscle. J. Gerontol. A. Biol. Sci. Med. Sci..

[56] Sakuma K, Yamaguchi A (2010). Molecular mechanisms in aging and current strategies to counteract sarcopenia. Curr. Aging Sci..

[57] Ziaaldini MM (2015). Exercise training increases anabolic and attenuates catabolic and apoptotic processes in aged skeletal muscle of male rats. Exp. Gerontol..

[58] Raue U, Slivka D, Jemiolo B, Hollon C, Trappe S (2006). Myogenic gene expression at rest and after a bout of resistance exercise in young (18–30 yr) and old (80–89 yr) women. J. Appl. Physiol..

[59] Léger B (2009). Atrogin-1, MuRF1, and FoXo, as well as phosphorylated GSK-3?? and 4E-BP1 are reduced in skeletal muscle of chronic spinal cord-injured patients. Muscle and Nerve.

[60] Greiwe JS, Bo C, Rubin DC, Yarasheski KE, Semenkovich CF (2001). Resistance exercise decreases skeletal muscle tumor necrosis factor alpha in frail elderly humans. FASEB J..

[61] Raue U, Jemiolo B, Yang Y, Trappe S (2015). TWEAK-Fn14 pathway activation after exercise in human skeletal muscle: insights from two exercise modes and a time course investigation. J. Appl. Physiol..

[62] McCarthy JJ, Esser KA (2010). Anabolic and catabolic pathways regulating skeletal muscle mass. Curr. Opin. Clin. Nutr. Metab. Care.

[63] Terzis G (2010). The degree of p70S6k and S6 phosphorylation in human skeletal muscle in response to resistance exercise depends on the training volume. Eur. J. Appl. Physiol..

[64] Barton-Davis ER, Shoturma DI, Sweeney HL (1999). Contribution of satellite cells to IGF-I induced hypertrophy of skeletal muscle. in. Acta Physiologica Scandinavica.

[65] Sacheck JM, Ohtsuka A, McLary SC, Goldberg AL (2004). IGF-I stimulates muscle growth by suppressing protein breakdown and expression of atrophy-related ubiquitin ligases, atrogin-1 and MuRF1. Am. J. Physiol. Endocrinol. Metab..

[66] Clemmons DR (2012). Metabolic Actions of Insulin-Like Growth Factor-I in Normal Physiology and Diabetes. Endocrinology and Metabolism Clinics of North America.

[67] de Cassia Marqueti R (2017). Resistance training minimizes the biomechanical effects of aging in three different rat tendons. J. Biomech..

[68] Goodpaster BH, He J, Watkins S, Kelley DE (2001). Skeletal muscle lipid content and insulin resistance: Evidence for a paradox in endurance-trained athletes. J. Clin. Endocrinol. Metab..

[69] Lo S, Russell JC (1970). & Taylor, a W. Determination of glycogen in small tissue samples. J. Appl. Physiol..

[70] Hill M, Goldspink G (2003). Expression and splicing of the insulin-like growth factor gene in rodent muscle is associated with muscle satellite (stem) cell activation following local tissue damage. J Physiol.

[71] Dheda K (2004). Validation of housekeeping genes for normalizing RNA expression in real-time PCR. Biotechniques.

