# Author Correction: Resistance training regulates gene expression of molecules associated with intramyocellular lipids, glucose signaling and fiber size in old rats

**DOI:** 10.1038/s41598-019-42462-w

**Published:** 2019-04-17

**Authors:** Manoel Benício Teixeira Ribeiro, Vinicius Guzzoni, Jeffrey M. Hord, Giselle Nunes Lopes, Rita de Cássia Marqueti, Rosângela Vieira de Andrade, Heloisa Sobreiro Selistre-de-Araujo, João Luiz Q. Durigan

**Affiliations:** 1 College of Physical Education, University of Brasília, Distrito Federal, Brazil; 2 Postdoctoral Fellowship, University of Brasília, Distrito Federal, Brazil; 3 Department of Molecular Physiology and Biophysics, Carver College of Medicine, University of Iowa, Iowa City, United States; 4 Department of Physiological Sciences, Center of Biological and Health Science, Federal University of São Carlos, São Carlos, Sao Paulo Brazil; 5 Graduate program of Rehabilitation Sciences, University of Brasilia, Distrito Federal, Brazil; 6 Graduate program of Genomics and Proteomics, Catholic University of Brasilia, Distrito Federal, Brazil

Correction to: *Scientific Reports* 10.1038/s41598-017-09343-6, published online 17 August 2017

The original version of this Article contained a typographical error in the spelling of the author Giselle Nunes Lopes, which was incorrectly given as Gisele Nunes Lopes. This has now been corrected in the PDF and HTML versions of the Article.

